# Evaluation of cuspid cortical anchorage with different sagittal patterns using cone-beam computed tomography: a retrospective study

**DOI:** 10.1186/s12903-023-02912-4

**Published:** 2023-04-15

**Authors:** Xiaoyu Wei, Yaqi Lin, Guanning Zhang, Jiawen Zheng, Lanxin Zhang, Yuqing Yang, Qing Zhao

**Affiliations:** 1 Orthodontic Centre, West China College of Stomatology, Sichuan University, 14#, 3rd Section, Renmin South Road, Chengdu, 610041 China; 2 State Key Laboratory of Oral Diseases, Sichuan University, 14#, 3rd Section, Renmin South Road, Chengdu, 610041 China

**Keywords:** Three-dimensional imaging, Cone-beam computed tomography, Alveolar bone, Cuspids, Skeletal malocclusion, Orthodontic treatment

## Abstract

**Background:**

No studies have focused on cortical anchorage resistance in cuspids, this study aimed to characterize the cortical anchorage according to sagittal skeletal classes using cone-beam computed tomography (CBCT).

**Methods:**

CBCT images of 104 men and 104 women were divided into skeletal class I, II, and III malocclusion groups. Skeletal and dental evaluations were performed on the sagittal and axial cross-sections. One-way analysis of variance followed by least significant difference post-hoc tests was used for group differences. Multiple linear regression was performed to evaluate the relationship between influential factors and cuspid cortical anchorage.

**Results:**

All cuspids were close to the labial bone cortex in different sagittal skeletal patterns and had different inclinations. There was a significant difference in the apical root position of cuspids in the alveolar bone; however, no significant difference in the middle or cervical portions of the root was found between different sagittal facial patterns. The middle of the cuspid root was embedded to the greatest extent in the labial bone cortex, with no significant difference between the sagittal patterns. For all sagittal patterns, 6.03 ± 4.41° (men) and 6.08 ± 4.45° (women) may be appropriate root control angles to keep maxillary cuspids’ roots detached from the labial bone cortex.

**Conclusions:**

Comparison of skeletal class I, II, and III malocclusion patients showed that dental compensation alleviated sagittal skeletal discrepancies in the cuspid positions of all patients, regardless of the malocclusion class. Detailed treatment procedures and clear treatment boundaries of cuspids with different skeletal patterns can improve the treatment time, periodontal bone remodeling, and post-treatment long-term stability. Future studies on cuspids with different dentofacial patterns and considering cuspid morphology and periodontal condition may provide more evidence for clinical treatment.

## Background

Orthodontics is dedicated to the precise control of tooth position in a three-dimensional (3D) orientation to achieve an ideal occlusal relationship with targeted tooth movement. This has led to the emergence of edgewise orthodontic systems, straight-arch orthodontic systems, and aligners [1, 2]. However, the evolution of orthodontic systems and materials has not fundamentally altered the orthodontic treatment philosophy of aligning the teeth, closing the extraction space under the 3D control of the teeth, and refining the occlusal relationship.

In clinical practice, anterior tooth intrusion and cuspid distal movement with either fixed orthodontic appliance systems or clear aligner systems are often difficult. Orthodontic tooth movement involves bone remodeling, with bone resorption on the pressure side and bone formation on the tension side, and is influenced by factors, such as the orthodontic force, bone density, and cortical anchorage [3, 4]. One of the key factors is the proximity of the tooth root to the cortical bone. In contrast to the porous structure of the cribriform plate and the less dense cancellous bone, the thin, hard layer of bone cortex provides sufficient resistance to tooth movement [5], and the bone cortex is often distributed in the direction of expected tooth movement, such as the labial bone cortex during intrusion of the anterior teeth, and the buccolingual bone cortex in the extraction area during closure of the extraction space. Appropriate torque settings are critical to avoid cortical anchorage. As the roots are generally submerged in the alveolar bone and invisible within the oral cavity, the tooth torque is often set based on visual inspection or experience, which may cause cortical anchorage and impede smooth and physiological orthodontic treatment.

To study the limits of tooth movement, some studies have focused on the thickness of the alveolar bone or the bone cortex [6, 7]. Additionally, studies have reported modulating alveolar bone remodeling in the incisal region after orthodontic treatment to overcome the limitations of orthodontic tooth movement [8]. Most patients with malocclusion have skeletal discrepancies, and the linked compensations of alveolar bone morphology and tooth angles increase the difficulty of diagnosis [9]. Moreover, different sagittal skeletal patterns are associated with different degrees of risk of dehiscence and fenestration [10]. Recent studies have therefore focused on the effect of different skeletal facial morphologies on the thickness of the alveolar bone in the anterior region or on the position of the anterior teeth in the alveolar bone [11–13]. However, most studies did not consider the cortical and cancellous bone separately and have focused on the incisors. The cuspids, unlike the incisors, have the longest and thickest roots, are located at the turn of the arch, and are subject to both sagittal and transverse orientation influences. Thus, the torque of the cuspids cannot be accurately determined by intraoral observation and two-dimensional (2D) panoramic radiography alone [14]. Cuspid cortical anchorage, a crucial factor in the alignment and closure of the extraction space, has rarely been studied. Identifying the inclination and position of the cuspids facilitates their physiological movement and avoidance of iatrogenic dehiscence or fenestration.

Cone-beam computed tomography (CBCT) has been widely used in dentistry because it provides more comprehensive information relative to 2D images [15]. To our knowledge, no study has demonstrated the position of cuspids in relation to the alveolar bone and the method of cuspid compensation in different sagittal skeletal patterns using 3D images. Therefore, this study aimed to analyze the characteristics of cuspid roots cortical anchorage in patients with different sagittal skeletal facial morphologies using CBCT, evaluate the potential skeletal and dental features that may influence the relationship between cuspid root and alveolar bone cortex, and investigate the relatively ideal root control angle that should be applied when the cuspids are detached from the bone cortex to provide a reference for orthodontists to set the cuspid position.

## Methods

This retrospective cross-sectional study was approved by the Ethics Committee of the National Key Laboratory of Oral Diseases and the Ethics Committee of the West China Stomatology Hospital of Sichuan University (protocol number: WCHSIRB-D-2020-399). The study sample was selected from patients who visited. We searched the hospital case system to screen for cases with CBCT examinations in the primary diagnosis data and collected participants according to the following inclusion and exclusion criteria. CBCT is performed at the discretion of the treating physician and is used to provide information that is insufficient to provide with 2D images of the temporomandibular joint, roots, and obstructed teeth, etc. We explained the purpose of our study in detail to the participants, and all participants understood and signed informed consent.

The inclusion criteria were as follows: (1) facial symmetry; (2) complete permanent dentition; (3) no posterior crossbite or scissors bite; (4) well-aligned maxillary and mandibular anterior teeth with fully developed cuspid roots and no significant root curvature (The severity of crowding is less than 2 mm); (5) no bone defects: the distance from the top of the alveolar ridge to the cemento-enamel junction (CEJ) of the cuspid should be less than 3 mm [16]; and (6) normodivergent facial patterns (22° ≤ Frankfort mandibular plane angle (FMA) ≤ 30°). The exclusion criteria were as follows: (1) history of orthodontic treatment; (2) pathological changes, such as periapical inflammation, cysts, tumors, and root resorption involving the cuspid region; and (3) systemic diseases. After screening, 208 patients (104 men and 104 women; 25.42 ± 6.24 years), with randomly selected unilateral 208 maxillary cuspids, were included in this study and divided into three groups according to the degree of ANB angle (The intersection angle of the line connecting the subspinale, nasion and supramental points) on cephalometric findings: skeletal class I malocclusion (n = 64, ANB angle between 1° and 4° and an angle class I molar relationship), skeletal class II malocclusion (n = 78, ANB angle greater than 4° and an angle class II molar relationship), and skeletal class III malocclusion (n = 66, ANB angle less than 1° and an angle class III molar relationship); the demographic data are shown in Table 1. The sample size was calculated based on a previous study using PASS software (version 15; Kaysville, Utah, USA) with 80% power of test and 0.05 significance level as reference [17].

**Table 1:** Demographic data of the subjects

	Class I		Class II		Class III	
	Men	Women	Men	Women	Men	Women
n	32	16	38	20	34	16
Age(y)	24.97 ± 5.72	26.84 ± 6.54	26.03 ± 7.68	26.98 ± 7.86	23.71 ± 3.15	23.63 ± 3.62
ANB(°)	2.38 ± 0.76	2.48 ± 0.76	6.29 ± 1.72	5.52 ± 1.25	−2.90 ± 3.28	2.00 ± 2.05
FMA(°)	24.66 ± 2.35	24.37 ± 2.06	24.75 ± 2.08	24.7 ± 2.10	23.85 ± 2.07	23.80 ± 1.18

All CBCT data were obtained using a CBCT machine (3D Accuitomo F170; J Morita Manufacturing, Kyoto, Japan) with exposure parameters of 4.5 mA and 85 kV, exposure time of 17.5 s, voxel size of 0.25 mm, and scan area of 140 × 100 mm. All digital lateral cephalometric were obtained by the same radiographer (Vatech PaX-I 2D, Gyeonggi, Korea). One investigator randomly numbered all CBCT and cephalometric data without patient information. The data were measured by two other investigators using Dolphin Imaging Software (version 11.9 premium, Dolphin Imaging & Management Solutions, Chatsworth, USA) and i-Dixel One Volume Viewer software (i-Dixel 3DX, Version 2.8, J Morita Mfg. Corp., Osaka, Japan).

**Fig. 1:**
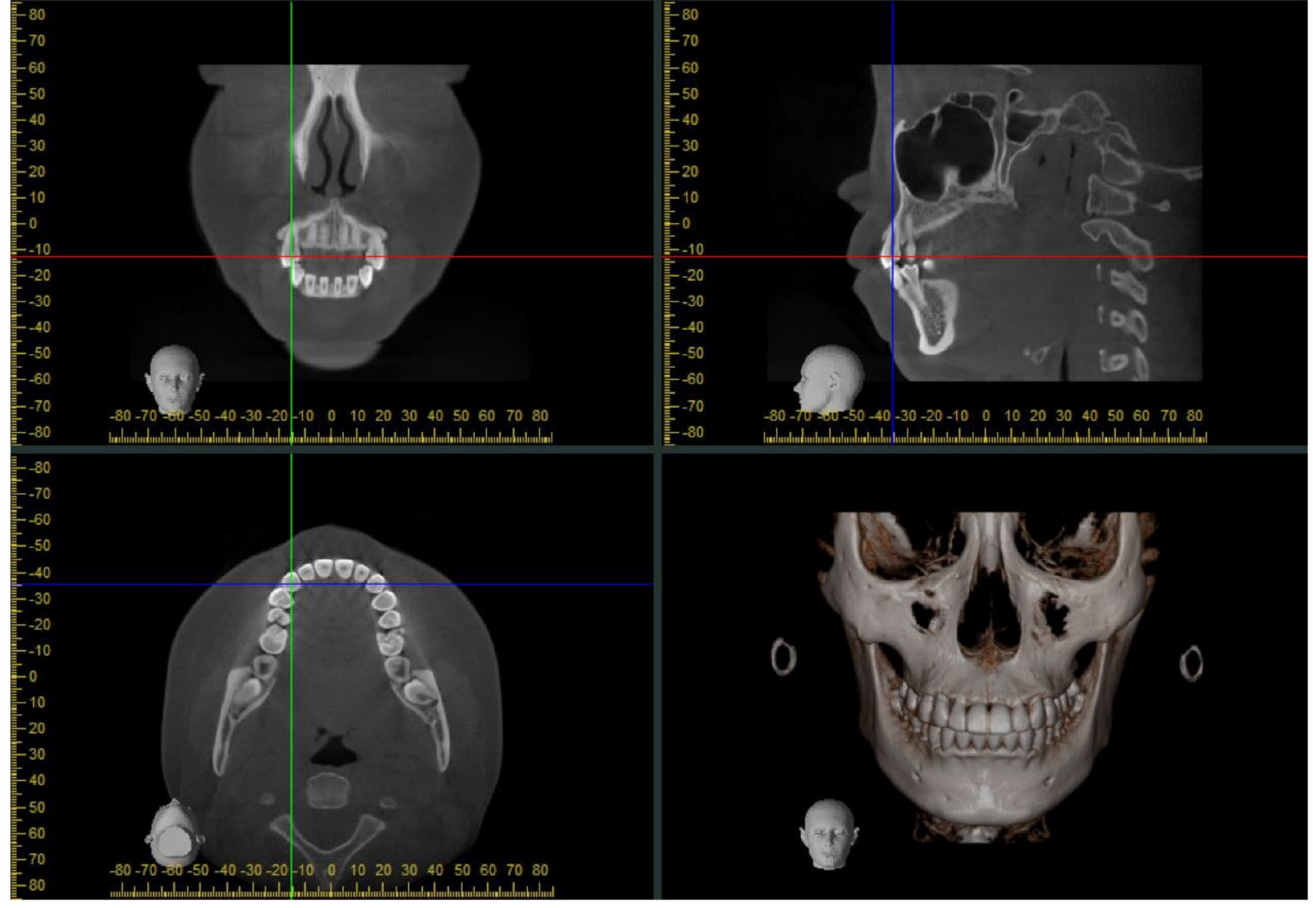
Three-dimensional image presentation of CBCT. The green guideline adjusts the sagittal orientation. The red guideline adjusts the axial orientation. The blue guideline adjusts the coronal orientation

**Fig. 2:**
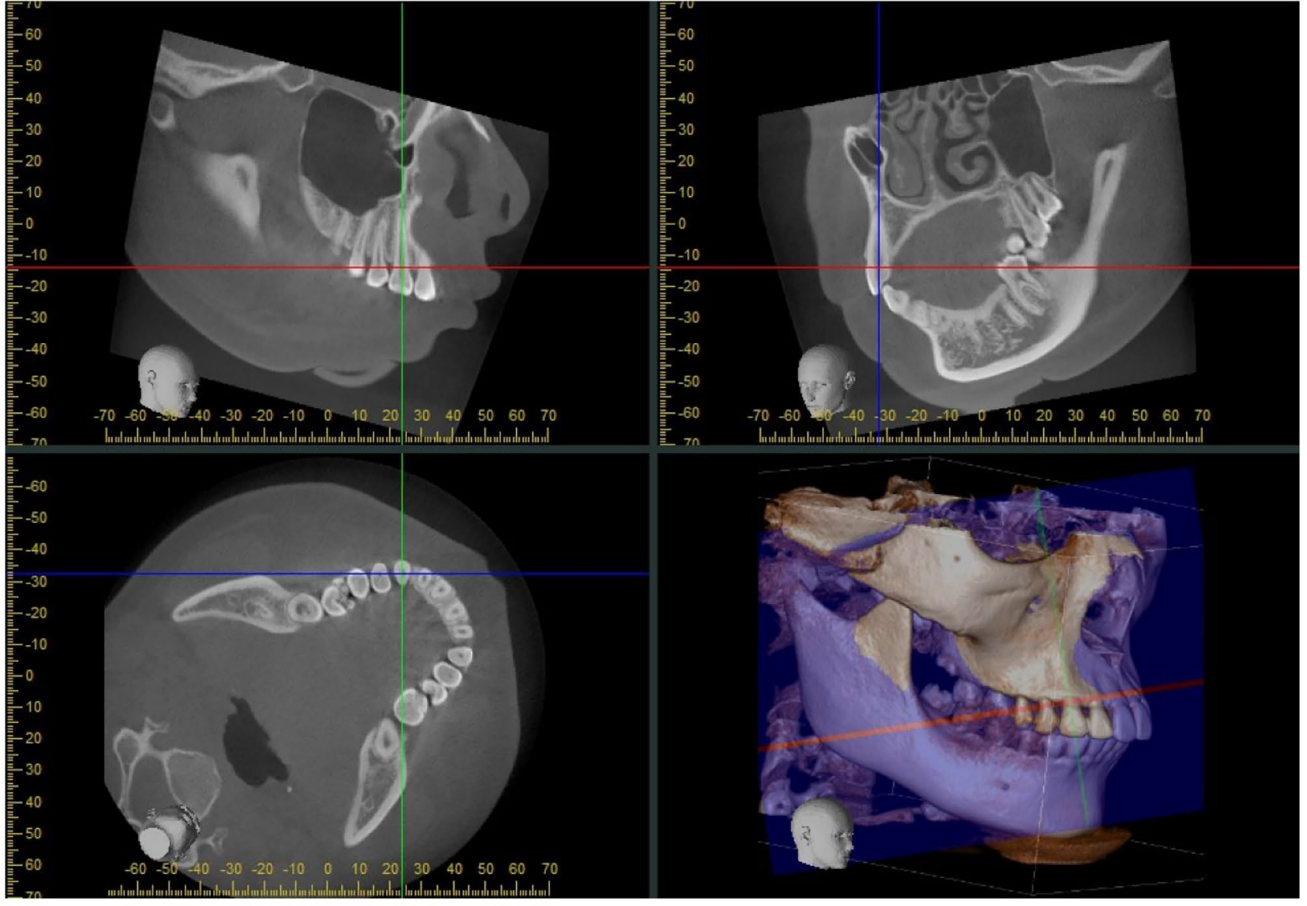
Presentation of the reference planes for the measurement of the cuspid

**Fig. 3:**
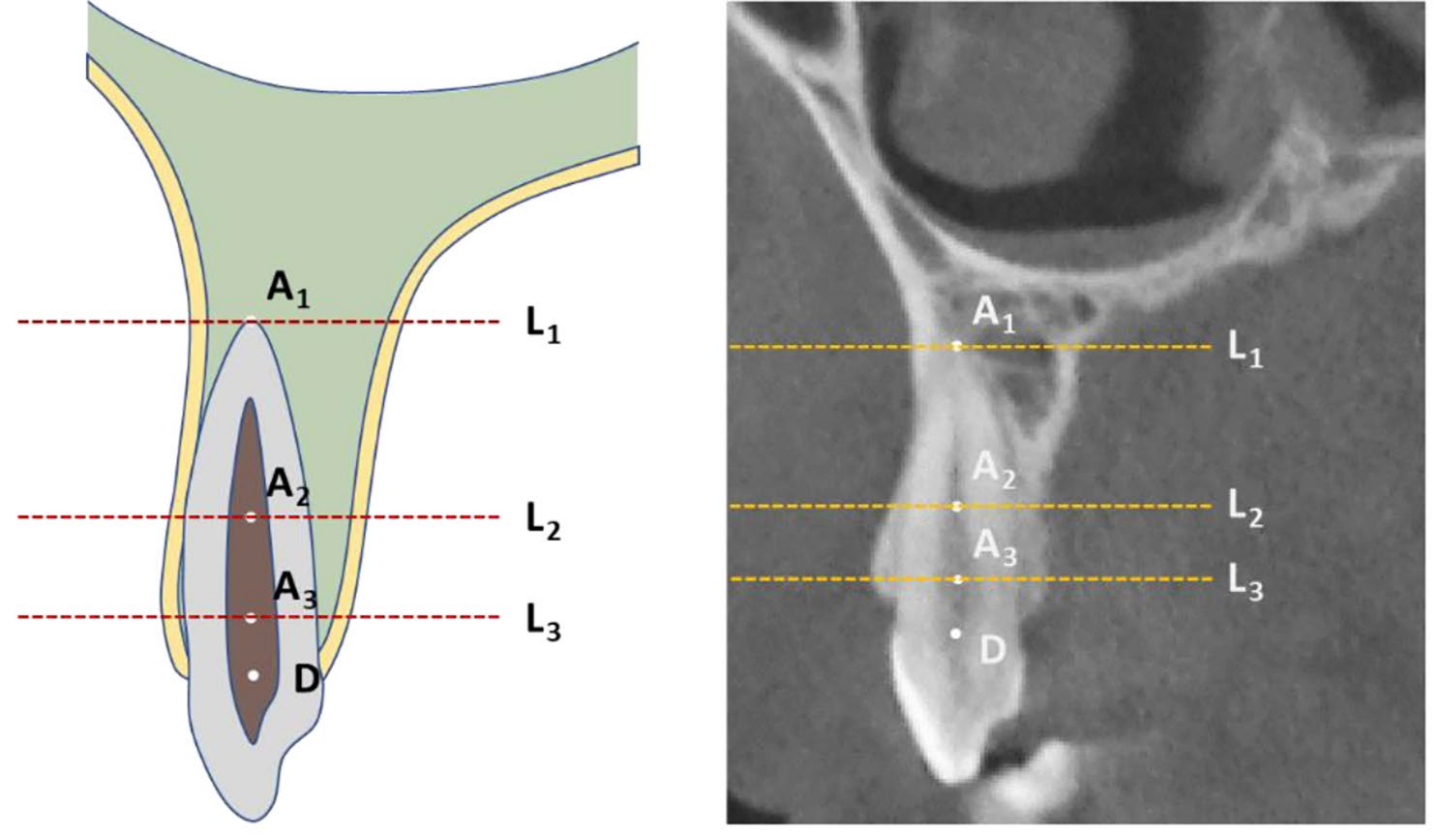
Three levels of the cuspid root. A_1_: the apical point of the cuspid; A_2_: the mid-root point of the cuspid; A_3_: the cervical-root of the cuspid; D: the intersection of the long axis of the cuspid and the CEJ; L_1_: the apical-root level; L_2_: the mid-root level; and L_3_: the cervical-root level

**Fig. 4:**
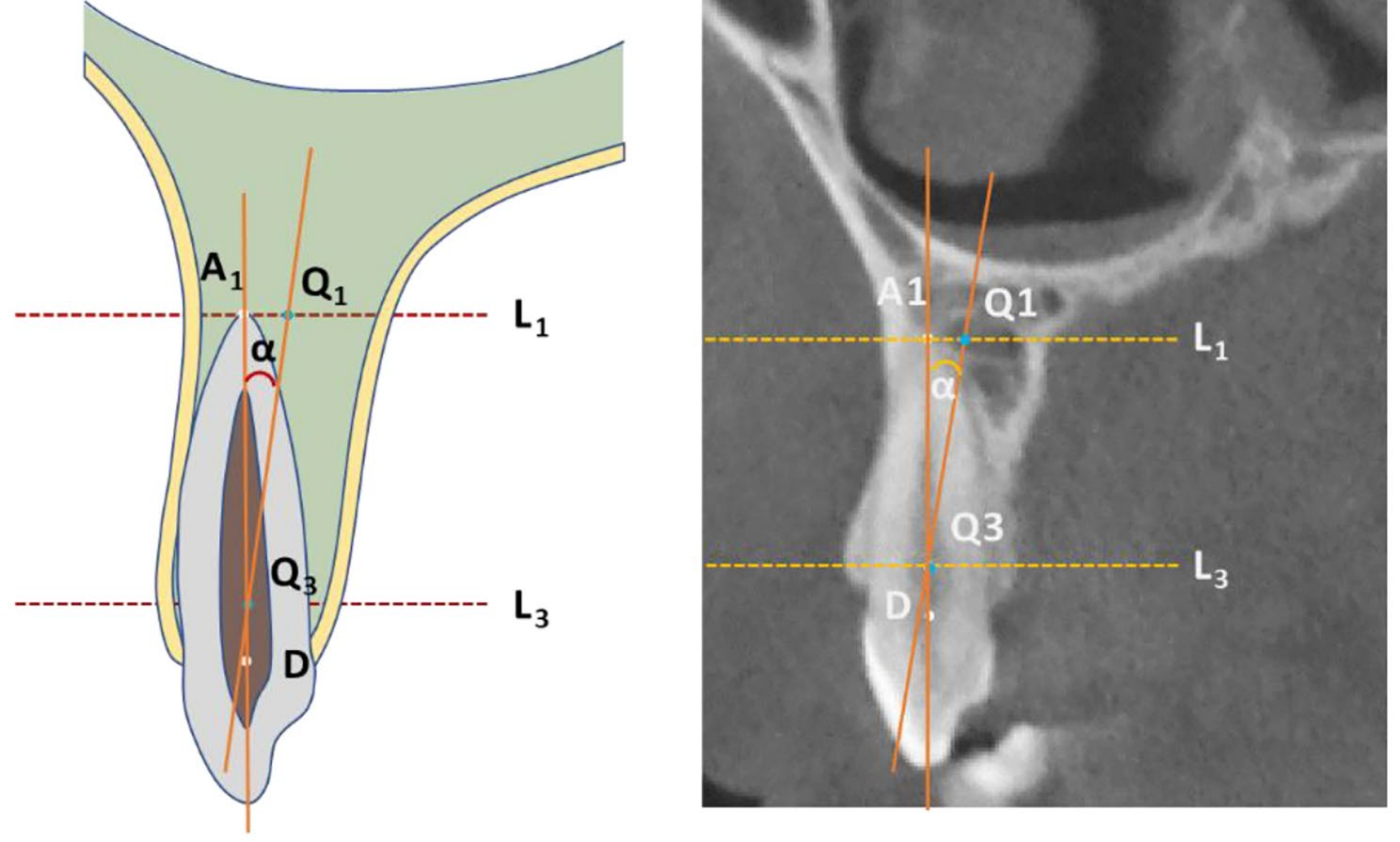
Inclination angle of the cuspid. Q_1_: the alveolar bone midpoint at the L_1_ level; Q_3_: the alveolar bone midpoint at the L_3_ level; and α: the inclination angle of the cuspid

**Fig. 5:**
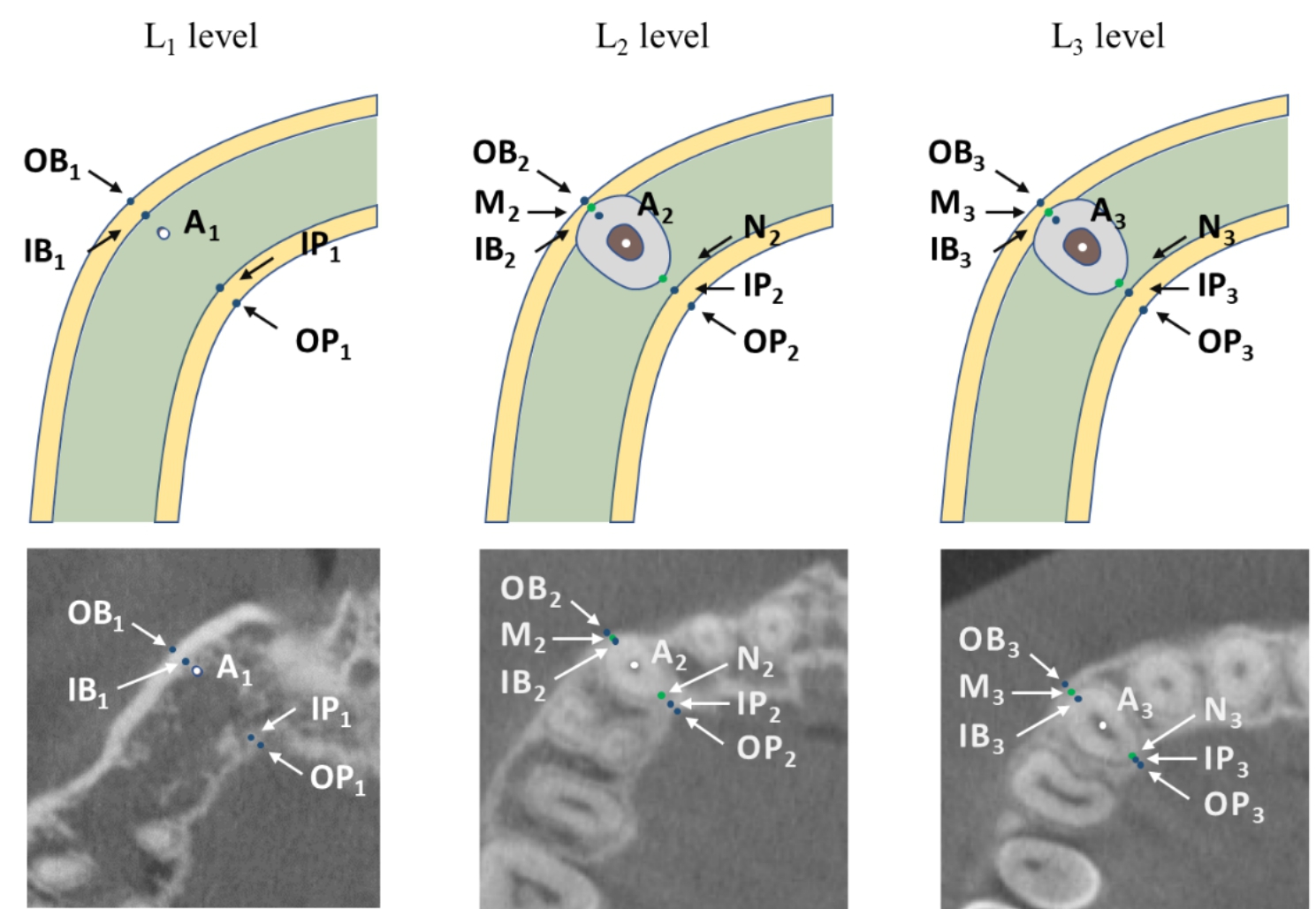
Marking points of the root and the alveolar. OB_1_: the outer edge of the labial bone cortex at the L_1_ level; IB_1_: the inner edge of the labial bone cortex at the L_1_ level; IP_1_: the inner edge of the palatal bone cortex at the L_1_ level; OP_1_: the outer edge of the palatal bone cortex at the L_1_ level; OB_2_: the outer edge of the labial bone cortex at the L_2_ level; IB_2_: the inner edge of the labial bone cortex at the L_2_ level; IP_2_: the inner edge of the palatal bone cortex at the L_2_ level; OP_2_: the outer edge of the palatal bone cortex at the L_2_ level; M_2_: the labial tangent point of the root at the L_2_ level; N_2_: the palatal tangent point of the root at the L_2_ level; OB_3_: the outer edge of the labial bone cortex at the L_3_ level; IB_3_: the inner edge of the labial bone cortex at the L_3_ level; IP_3_: the inner edge of the palatal bone cortex at the L_3_ level; OP_3_: the outer edge of the palatal bone cortex at the L_3_ level; M_3_: the labial tangent point of the root at the L_3_ level; and N_3_: the palatal tangent point of the root at the L_3_ level

**Fig. 6:**
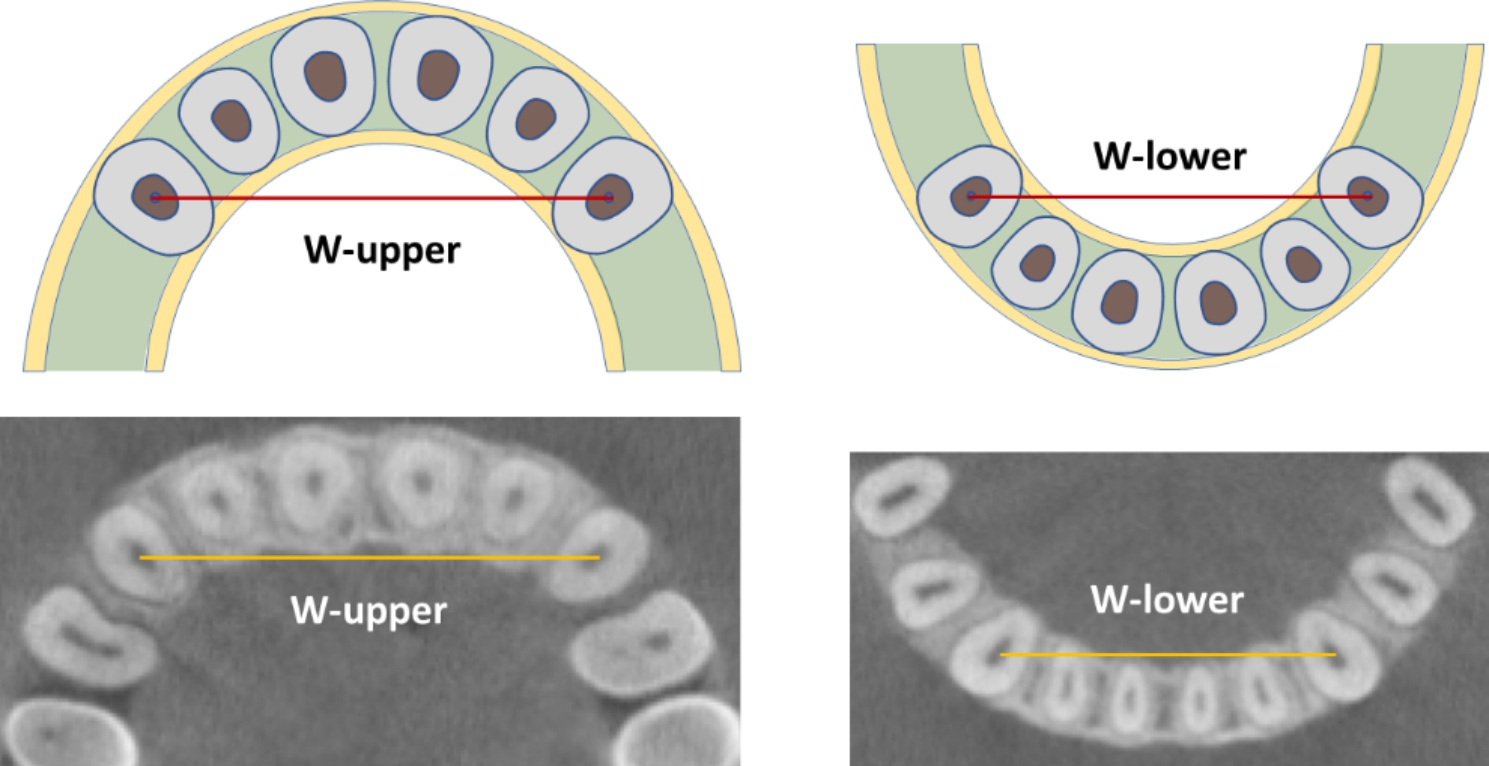
Maxillary and mandibular inter-cuspid alveolar bone width. W-upper: the maxillary inter-cuspid alveolar bone width; W-lower: the mandibular inter-cuspid alveolar bone width

**Fig. 7:**
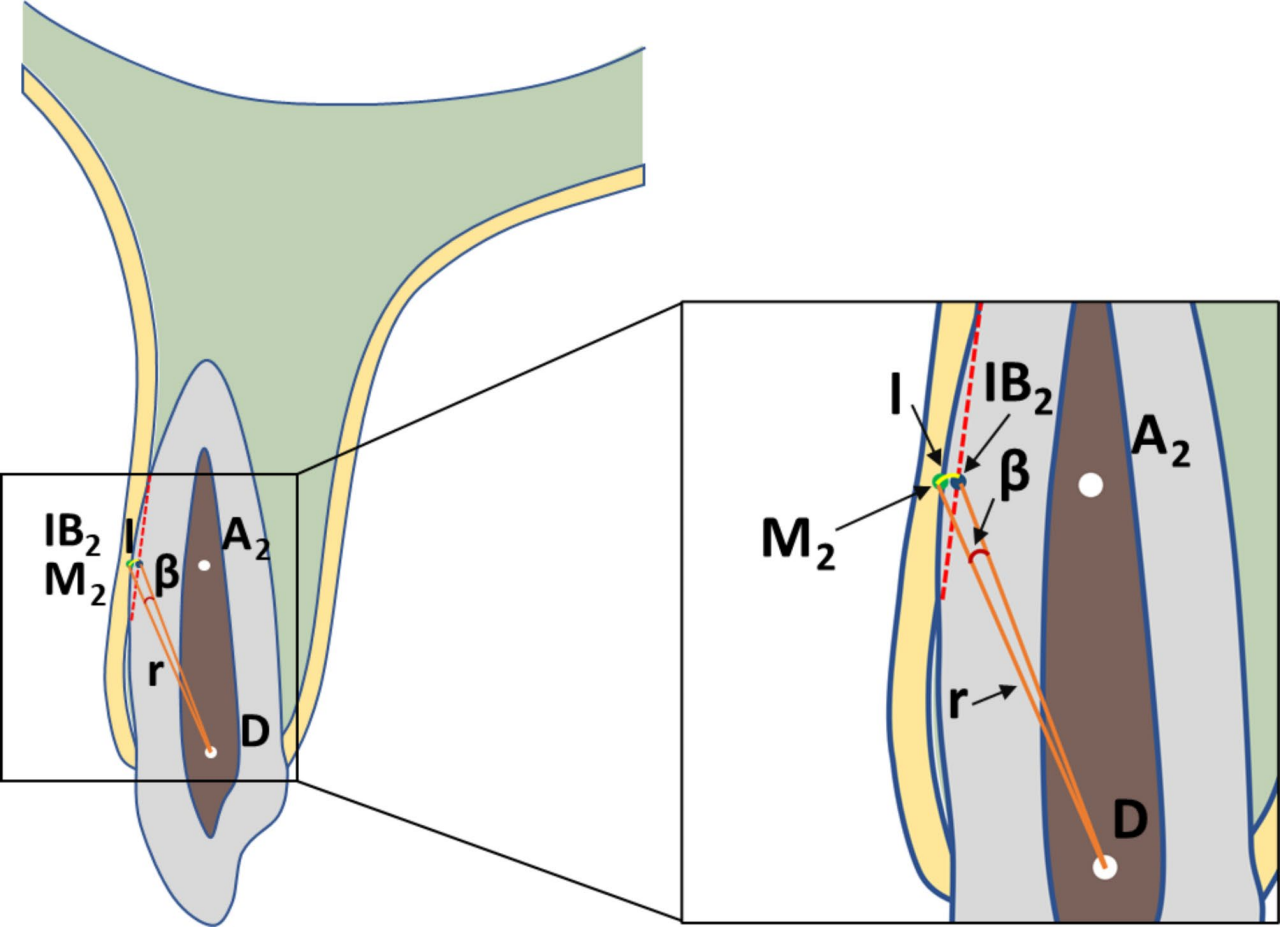
Ideal root control angle of maxillary cuspids to keep the roots detached from the labial bone cortex. β: the ideal root control angle; r = M_2_-D = ((A_2_-D)^2^ + (A_2_-M_2_)^2^) ^½^; l = 2πr × β/360 ≈ M_2_-IB_2_; and β ≈ (M_2_-IB_2_) × 360 / 2πr

The 3D spatial position of the cuspids was represented by three mutually perpendicular planes and the reconstructed image in the i-Dixel software (Fig. 1). All measurements were performed in the maximal labiolingual direction of each tooth, a method validated in previous studies [18]. As shown in Fig. 2, the green guideline crossed the maximum labial-palatal diameter of the cuspid in the axial plane, and the green and blue guidelines passed through the cusp and apical points of the cuspid in the coronal and sagittal planes, separately. A_1_, the mid root point (A_2_), and a point 4 mm below the CEJ (A_3_) helped determine the three measurement planes L_1_, L_2_, and L_3_ (Fig. 3). The alveolar bone midpoints (Q_1_ and Q_3_) in the L_1_ and L_3_ sections were connected to determine the direction of the alveolar bone inclination, and the cuspid inclination angle (α) was the angle at which the alveolar bone inclination line and long axis of the cuspid intersected (Fig. 4). The cuspid and alveolar landmarks in the axial plane at three levels are illustrated in Fig. 5. The center points of the pulp chambers of the maxillary and mandibular left and right cuspids were used to measure the maxillary and mandibular intercuspid alveolar bone width (W-upper and W-lower) (Fig. 6). To calculate the rotation angle (β) of the cuspid detached from the labial bone cortex with D as the center of rotation, the radius of rotation r = M_2_-D = ((A_2_-D) ^2^ + (A_2_-M_2_) ^2^) ^1/2^ was calculated using the Pythagorean theorem. According to the arc length formula, arc l = 2πr × β/360. Given that l is much smaller than the radius r, for simplicity of calculation, l was set to approximately M_2_-IB_2_, and then β ≈ (M_2_-IB_2_) × 360 / 2πr (Fig. 7). All CBCT measurement data were analyzed using a scale with an accuracy level of 0.01 mm. The cuspid, alveolar, and measuring plane landmarks are defined in Table 2, and all measurements are presented in Table 3. The value was negative if the cuspid root landmarks were farther from the center of the alveolar bone than the alveolar bone landmarks.

**Table 2:** Landmarks and definitions based on two-dimensional coordinates

Landmarks		Definitions
Level		
	L_1_	The apical root level of the cuspid
	L_2_	The mid root level of the cuspid
	L_3_	The cervical root level of the cuspid
Root		
	A_1_	The apical point of the cuspid
	A_2_	The mid root point of the cuspid
	A_3_	The 4 mm point below the CEJ of the cuspid
	D	The intersection of the long axis of the cuspid and the CEJ
	M_2_	The labial tangent point of the root at the L_2_ level
	N_2_	The palatal tangent point of the root at the L_2_ level
	M_3_	The labial tangent point of the root at the L_3_ level
	N_3_	The palatal tangent point of the root at the L_3_ level
Alveolar		
	Q_1_	The alveolar bone midpoint of the L_1_ section
	Q_3_	The alveolar bone midpoint of the L_3_ section
	OB_1_	The outer edge of the labial bone cortex at the L_1_ level
	OB_2_	The outer edge of the labial bone cortex at the L_2_ level
	OB_3_	The outer edge of the labial bone cortex at the L_3_ level
	IB_1_	The inner edge of the labial bone cortex at the L_1_ level
	IB_2_	The inner edge of the labial bone cortex at the L_2_ level
	IB_3_	The inner edge of the labial bone cortex at the L_3_ level
	OP_1_	The outer edge of the palatal bone cortex at the L_1_ level
	OP_2_	The outer edge of the palatal bone cortex at the L_2_ level
	OP_3_	The outer edge of the palatal bone cortex at the L_3_ level
	IP_1_	The inner edge of the palatal bone cortex at the L_1_ level
	IP_2_	The inner edge of the palatal bone cortex at the L_2_ level
	IP_3_	The inner edge of the palatal bone cortex at the L_3_ level

**Table 3:** Measured variables between the alveolar bone and dental landmarks

Abbreviation	Definition
A_1_-OB_1_	The distance between A_1_ to OB_1_
A_1_-IB_1_	The distance between A_1_ to IB_1_
A_1_-OP_1_	The distance between A_1_ to OP_1_
A_1_-IP_1_	The distance between A_1_ to IP_1_
A_2_-M_2_	The distance between A_2_ to M_2_
A_2_-IB_2_	The distance between A_2_ to IB_2_
A_2_-OB_2_	The distance between A_2_ to OB_2_
A_2_-N_2_	The distance between A_2_ to N_2_
A_2_-IP_2_	The distance between A_2_ to IP_2_
A_2_-OP_2_	The distance between A_2_ to OP_2_
A_3_-M_3_	The distance between A_3_ to M_3_
A_3_-IB_3_	The distance between A_3_ to IB_3_
A_3_-OB_3_	The distance between A_3_ to OB_3_
A_3_-N_3_	The distance between A_3_ to N_3_
A_3_-IP_3_	The distance between A_3_ to IP_3_
A_3_-OP_3_	The distance between A_3_ to OP_3_
OB_1_-OP_1_	The apical root alveolar bone thickness
OB_1_-IB_1_	The apical root labial bone cortical thickness
OP_1_-IP_1_	The apical root palatal bone cortical thickness
OB_2_-OP_2_	The mid root alveolar bone thickness
OB_2_-IB_2_	The mid root labial bone cortical thickness
OP_2_-IP_2_	The mid root palatal bone cortical thickness
OB_3_-OP_3_	The cervical root alveolar bone thickness
OB_3_-IB_3_	The cervical root labial bone cortical thickness
OP_3_-IP_3_	The cervical root palatal bone cortical thickness
α	The cuspid inclination angle
r	The radius of rotation
β	The rotation angle of the cuspid detached from the labial bone cortex with D as the center of rotation
W-upper	The maxillary intercuspid alveolar bone width
W-lower	The mandibular intercuspid alveolar bone width
WUL	The distance between W-upper and W-lower

Before the measurements, two investigators were trained by a professor of orthodontics and a professor of radiology with extensive experience to improve the measurement accuracy. Then, 20% of the cephalometric lateral radiographs and CBCT data were randomly selected, and each investigator measured the data twice, with a one-week interval between each measurement. Intragroup and intergroup correlation coefficient analyses were used to test the intraobserver and interobserver consistency. Normality tests were performed using the Shapiro-Wilk test. One-way analysis of variance followed by least significant difference post-hoc tests was used to compare the differences between different group measures. Skeletal and dental characteristics including ANB, FMA, U1-SN (Posterior inferior intersection angle between the long axis of the upper central incisor and the SN plane), W-upper, W-lower, WUL and α were selected in stepwise multiple linear regression, which was conducted to assess potential characteristics that may have an effect on A_1_-IB_1_, M_2_-IB_2_ and M_3_-IB_3_. All statistical analyses were conducted using IBM SPSS Statistics for Windows (Version 26.0; IBM Corporation, Armonk, New, USA). Statistical significance was set at P < 0.05.

## Results

In this study, the intergroup and intragroup coefficients of all variables are greater than 0.95, which show excellent intergroup and intragroup reproducibility of the measurements.

Table 4 shows the alveolar bone thickness and labial and lingual bone cortical thickness at apical, middle, and cervical root levels in patients with skeletal class I, class II, and class III malocclusion. There were no significant differences between the three malocclusion classes.

**Table 4:** Comparison of the alveolar bone and bone cortical thickness in different skeletal facial patterns

		Class I	Class II	Class III	P value
Variables		Mean ± SD	Mean ± SD	Mean ± SD	
L_1_ level					
OB_1_-OP_1_	Men	10.74 ± 1.92	11.43 ± 2.51	11.21 ± 2.26	0.447
	Women	11.49 ± 2.49	10.85 ± 2.66	11.36 ± 1.80	0.475
OB_1_-IB_1_	Men	1.49 ± 0.29	1.62 ± 0.31	1.56 ± 0.32	0.215
	Women	1.52 ± 0.36	1.66 ± 0.32	1.58 ± 0.37	0.209
OP_1_-IP_1_	Men	1.39 ± 0.52	1.46 ± 0.55	1.44 ± 0.59	0.782
	Women	1.43 ± 0.72	1.54 ± 0.66	1.36 ± 0.50	0.478
L_2_ level					
OB_2_-OP_2_	Men	9.61 ± 1.34	9.63 ± 1.39	9.82 ± 1.72	0.815
	Women	9.88 ± 0.97	9.38 ± 11.68	9.86 ± 1.73	0.277
OB_2_-IB_2_	Men	1.04 ± 0.40	1.02 ± 0.36	1.15 ± 0.60	0.468
	Women	1.09 ± 0.58	1.01 ± 0.36	1.10 ± 0.66	0.730
OP_2_-IP_2_	Men	1.83 ± 0.54	1.73 ± 0.54	1.76 ± 0.43	0.686
	Women	1.86 ± 0.43	1.73 ± 0.65	1.83 ± 0.48	0.554
L_3_ level					
OB_3_-OP_3_	Men	7.73 ± 1.40	7.71 ± 1.21	7.77 ± 1.79	0.985
	Women	7.39 ± 1.45	7.83 ± 1.28	7.95 ± 1.67	0.269
OB_3_-IB_3_	Men	1.02 ± 0.33	1.03 ± 0.34	1.03 ± 0.36	0.991
	Women	0.97 ± 0.31	1.01 ± 0.33	1.11 ± 0.37	0.248
OP_3_-IP_3_	Men	1.39 ± 0.45	1.41 ± 0.42	1.26 ± 0.46	0.301
	Women	1.29 ± 0.36	1.27 ± 0.26	1.32 ± 0.48	0.822

^*^P is significant at 0.05.

^†^P is significant at 0.01.

SD, standard deviation.

Table 5 shows the position of the cuspid roots relative to the alveolar bone in the different skeletal facial types. At the apical root level, cuspid roots were close to the labial bone cortex in both sexes in class II. The apical portion of the roots was farther away from the labial bone cortex in skeletal class I of women and class III of men malocclusion than in class II of both sexes, with significant difference. At the middle and cervical root levels, there were no significant differences in the position of the cuspid roots in any of the groups. However, it should be noted that at the middle root level, the roots of the cuspids touched the labial bone cortex and were close to the outer edge of the labial bone cortex. At the cervical root level, the roots of the cuspids still touched the labial bone cortex.

**Table 5:** Position of the cuspid roots in relation to the alveolar bone in different skeletal facial patterns

		Class I	Class II	Class III	P value
Variables		Mean ± SD	Mean ± SD	Mean ± SD	
L_1_ level					
A_1_-IB_1_	Men	0.63 ± 0.83	0.04 ± 0.76	1.10 ± 0.90	<0.001^†^
	Women	0.93 ± 1.04	−0.11 ± 0.77	0.77 ± 0.79	<0.001^†^
A_1_-OB_1_	Men	2.12 ± 0.88	1.66 ± 0.81	2.66 ± 0.92	<0.001^†^
	Women	2.45 ± 1.23	1.77 ± 0.77	2.35 ± 0.81	0.005^†^
A_1_-IP_1_	Men	7.25 ± 1.65	8.31 ± 2.57	7.11 ± 2.32	0.050
	Women	7.61 ± 2.18	7.53 ± 2.59	7.65 ± 2.03	0.977
A_1_-OP_1_	Men	8.63 ± 1.83	9.77 ± 2.65	8.54 ± 2.12	0.039^*^
	Women	9.03 ± 2.37	9.07 ± 2.79	9.01 ± 1.90	0.994
L_2_ level					
M_2_-IB_2_	Men	−0.92 ± 0.59	−0.77 ± 0.58	−1.06 ± 0.82	0.188
	Women	−1.07 ± 0.59	−0.81 ± 0.58	−0.88 ± 0.84	0.252
M_2_-OB_2_	Men	0.12 ± 0.66	0.26 ± 0.70	0.09 ± 0.83	0.597
	Women	0.02 ± 0.63	0.20 ± 0.59	0.22 ± 1.02	0.524
N_2_-IP_2_	Men	1.58 ± 0.97	1.41 ± 1.09	1.45 ± 1.06	0.779
	Women	1.75 ± 0.84	1.30 ± 0.99	1.35 ± 1.02	0.120
N_2_-OP_2_	Men	3.42 ± 1.18	3.14 ± 1.10	3.21 ± 1.24	0.604
	Women	3.61 ± 1.02	3.04 ± 1.22	3.19 ± 1.15	0.103
L_3_ level					
M_3_-IB_3_	Men	−0.16 ± 0.62	−0.25 ± 0.50	−0.34 ± 0.73	0.506
	Women	−0.31 ± 0.58	−0.17 ± 0.52	−0.29 ± 0.76	0.543
M_3_-OB_3_	Men	0.87 ± 0.64	0.78 ± 0.51	0.69 ± 0.67	0.497
	Women	0.66 ± 0.64	0.84 ± 0.48	0.82 ± 0.70	0.410
N_3_-IP_3_	Men	0.23 ± 0.59	0.13 ± 0.82	0.24 ± 0.60	0.750
	Women	0.47 ± 0.58	0.18 ± 0.60	0.17 ± 0.59	0.065
N_3_-OP_3_	Men	1.62 ± 0.70	1.54 ± 0.81	1.50 ± 0.77	0.795
	Women	1.76 ± 0.66	1.44 ± 0.65	1.50 ± 0.74	0.127

^*^P is significant at 0.05.

^†^P is significant at 0.01.

SD, standard deviation.

Table 6 compares the position of the cuspid roots relative to the labial bone cortex at different levels in patients with the same malocclusion type. The roots of the cuspids at the middle root level were located to the greatest extent in the labial bone cortex for all skeletal malocclusion types.

**Table 6:** Comparison of the distance between the roots of cuspids and the inner and outer edge of labial bone cortex at the different root levels

		L_1_ level: A_1_-IB_1_	L_2_ level: M_2_-IB_2_	L_3_ level: M_3_-IB_3_	P value	P value		
		Mean ± SD	Mean ± SD	Mean ± SD		L_1_ VS L_2_	L_2_ VS L_3_	L_1_VS L_3_
Class I	Men	0.63 ± 0.83	−0.92 ± 0.59	−0.16 ± 0.62	< 0.001^†^	< 0.001^†^	< 0.001^†^	< 0.001^†^
	Women	0.93 ± 1.04	−1.06 ± 0.59	−0.31 ± 0.58	< 0.001^†^	< 0.001^†^	< 0.001^†^	< 0.001^†^
Class II	Men	0.04 ± 0.76	−0.77 ± 0.58	−0.25 ± 0.50	< 0.001^†^	< 0.001^†^	< 0.001^†^	0.044^*^
	Women	0.11 ± 0.77	−0.81 ± 0.58	−0.17 ± 0.52	< 0.001^†^	< 0.001^†^	< 0.001^†^	0.054
Class III	Men	1.10 ± 0.90	−1.06 ± 0.82	−0.34 ± 0.73	< 0.001^†^	< 0.001^†^	< 0.001^†^	< 0.001^†^
	Women	0.77 ± 0.79	−0.88 ± 0.84	−0.29 ± 0.76	< 0.001^†^	< 0.001^†^	0.004^†^	< 0.001^†^

^*^P is significant at 0.05.

^†^P is significant at 0.01.

SD, standard deviation.

Table 7 shows no significant difference in root length and ideal root control angle among the different class groups. The mean ideal root control angle was 6.03 ± 4.41° in men and 6.08 ± 4.45° in women.

**Table 7:** Ideal root control angle to keep the roots of the maxillary cuspids detached from the labial bonebone cortex

		Class I	Class II	Class III	total		P value
Variables		Mean ± SD	Mean ± SD	Mean ± SD	Mean ± SD	n	
Root length (mm)	Men	15.38 ± 1.61	16.07 ± 2.14	16.03 ± 2.02	15.85 ± 1.96	104	0.275
	Women	15.61 ± 2.30	15.85 ± 2.19	15.70 ± 1.82	15.73 ± 1.82	104	0.787
M_2_-IB_2_ (mm)	Men	−0.92 ± 0.59	−0.77 ± 0.58	−1.06 ± 0.82	−0.91 ± 0.68	104	0.188
	Women	−1.07 ± 0.59	−0.81 ± 0.58	−0.88 ± 0.84	−0.91 ± 0.68	104	0.252
R (mm)	Men	8.29 ± 0.76	8.64 ± 1.01	8.69 ± 0.85	8.55 ± 0.90	104	0.138
	Women	8.40 ± 1.04	8.54 ± 1.02	8.52 ± 0.81	8.49 ± 0.96	104	0.807
Ideal root control angle (°)	Men	6.32 ± 4.01	5.02 ± 3.78	6.88 ± 5.27	6.03 ± 4.41	104	0.184
	Women	7.26 ± 4.07	5.41 ± 3.70	5.72 ± 5.47	6.08 ± 4.45	104	0.186

^*^P is significant at 0.05.

^†^P is significant at 0.01.

SD, standard deviation.

Table 8 demonstrates the factors influencing the cortical anchorage of cuspids by stepwise multiple linear regression. In general, cross-sectional indicators including W-lower and WUL; vertical indicators including FMA; and sagittal indicators including U_1_-SN and ANB all had an effect on the cortical anchorage of the cuspids. Only α, an indicator related to both cross-sectional and sagittal directions, were stable significant variables in A_1_-IB_1_, M_2_-IB_2_, and M_3_-IB_3_.

**Table 8:** Multiple linear regression demonstrating the relationship between cortical anchorage of cuspids and skeletal and dental characteristics

	β	Std. Error	P value
**A**_**1**_ **-IB**_**1**_			
men			
ANB (°)	−0.072^†^	0.018	< 0.001
FMA (°)	−0.075^*^	0.033	0.026
α (°)	−0.054^†^	0.011	< 0.001
women			
ANB (°)	−0.134^†^	0.027	< 0.001
FMA (°)	0.100^*^	0.040	0.014
W-lower (mm)	−0.095^*^	0.048	0.048
WUL (mm)	0.127^†^	0.047	0.008
α (°)	−0.073^†^	0.011	< 0.001
**M**_**2**_ **-IB**_**2**_			
men			
U_1_-SN (°)	−0.025^†^	0.007	0.001
women			
α (°)	0.021^*^	0.009	0.028
**M**_**3**_ **-IB**_**3**_			
men			
FMA (°)	0.083^†^	0.027	0.003
women			
α (°)	0.021^*^	0.009	0.028

^*^P is significant at 0.05.

^†^P is significant at 0.01.

Std. Error, standard error.

## Discussion

In our study, no differences were found in the alveolar bone thickness, labial bone cortical thickness, and lingual bone cortical thickness in the cuspid region of patients with skeletal class I, II, and III malocclusions. This indicates that the sagittal skeletal discrepancy did not significantly affect alveolar bone thickness in normodivergent facial patterns at different cuspid levels. The mean labial cortical thickness in cuspids ranged from 1.16 to 1.98 mm, similar to the findings of Shen et al., who found that the labial cortical distance of the maxillary cuspids ranged from 0.5 to 1.5 mm [19]. At the middle and cervical root levels, the thickness of the lingual bone cortex was more significant than that of the labial bone cortex, which was consistent with the findings of Lee et al. and Januario et al. [20, 21].

Compared to the labial bone cortex, lingual fenestration and dehiscence rarely occur in anterior teeth [22]. In our study, cuspids of all skeletal classes were in close proximity to the labial bone plate, which makes the cuspids very sensitive to fenestration and dehiscence caused by improper directions of tooth movement. Fenestration and dehiscence in the cuspid region may be associated with thinner buccal bone plates, pressure during the masticatory cycle, and a history of previous orthodontic treatment with nonphysiological tooth movement, excessive orthodontic forces, and compromised periodontal tissue integrity [23–26]. Characterization of the position of the cuspids relative to the alveolar bone helps reduce the risk of dehiscence and fenestration [27]. The root of the cuspids closer to the labial cortex indicated that cuspids were physiologically close to the labial bone plate, which may be closely related to the function of cuspids in tearing and cutting food and supporting the corners of the mouth [28]. At the apical root level, the mean A_1_-IB_1_ was the smallest and even negative in women with skeletal class II malocclusion, indicating that the apical roots of some class II cuspids were located outside the cancellous bone. This suggests that it is hazardous to apply a positive torque to the cuspid roots in skeletal class II malocclusion as it may lead to fenestration in the apical region. At the cervical root level, the roots of teeth contacted the labial bone cortex in all skeletal malocclusion types and were at risk of bone dehiscence if the cuspids were moving labially. Consistent with our study, studies have found an increase in dehiscence around cuspids after orthodontic treatment [29].

In skeletal class II malocclusion with a deep overbite, we generally first protrude the maxillary and mandibular incisors and then intrude them to avoid anterior alveolar fenestration and dehiscence. The cuspids are positioned close to the incisors and both are often intruded and moved distally at the same time. Therefore, we envisioned the possibility of investigating root control angles of the cuspids to achieve programmed movement similar to that of the incisors. Our study showed that in all skeletal malocclusion groups, the middle part of the root was mostly located in the labial bone cortex. Owing to the large variability in surface morphology and crown length, such as susceptibility to abrasion, the clinical crown center was not chosen, and the midpoint of the CEJ was considered the reference point. As a result, the estimated lingual torque applied was 6.03 ± 4.41° in men and 6.08 ± 4.45° in women. Unfortunately, the large variance reflects an insufficient sample size and within-sample heterogeneity, resulting in limited guidance for clinicians; however, the results suggest that at least positive labial torque should be prudently applied to cuspid roots in general. The importance of careful imaging or oral examination to avoid incorrect treatment on cuspids cannot be ignored.

Cuspid compensation complied with skeletal discrepancy. A recent meta-analysis showed that the relationship between alveolar bone thickness and age had no specific pattern; therefore, age was not considered as a crucial assessment factor in this study and was balanced at baseline [30]. In our study, ANB angle and α were negatively correlated with A_1_-IB_1_. Therefore, in patients with a large ANB angle or α, it is important to be aware of the risk of fenestration in the apical region of the cuspid. The effect of FMA on cortical anchorage in cuspids was not clear, suggesting that it may be affected by sagittal skeletal malalignment. M_2_-IB_2_, M_3_-IB_3_ had fewer influencing factors than A_1_-IB_1_, probably due to the significant narrowing of the root width of the cuspid from the middle to the apical part of the root, resulting in a greater sensitivity of A_1_-IB_1_ to skeletal and dental changes.

Characterization of the position of the cuspids relative to the alveolar bone also helps improve orthodontic efficiency [27]. In clear aligner treatment, in addition to the anatomical limitations, poor torque and step settings can also lower its efficiency in inducing cuspid movements (from 20 to 40%) [31]. In the staging design of invisible orthodontics, it may be advisable to adjust the root position of the cuspids in cancellous bone before intruding them or moving them distally, such as moving the root lingually first. In fixed orthodontics, the inappropriateness of the bracket preset torque and inaccuracy of bracket bonding would affect the achievement of the ideal torque of the cuspids [32]. The currently used MBT, ROTH, and edgewise straight wire arch orthodontic systems have preset torques of − 2°, − 7°, and 0° for cuspids, respectively. According to our findings, preset negative torque on cuspids in their original position could lead to cortical anchorage of the cuspids, and subsequently, adversely affect the adjacent teeth, lead to stagnation, or induce root resorption during orthodontic treatment.

Our study has some limitations. First, for the same inner skeletal pattern, our study did not reveal whether arch form affected the position of the cuspids. Second, the observations in the three defined planes cannot truly depict the relationship between the root morphology of cuspids and the bone cortex. The portion of cuspids located within the bone cortex may lie between the three levels. The determination of this level depends on improving CBCT accuracy, advancement of image recognition technology, optimization of intuitive artificial intelligence fixed-point curve fitting, and accumulation of data volume. Moreover, we included only normodivergent patients with well aligned cuspid, and the generalized application of the findings is limited. Imaging or oral examination, considerations of biology and biomechanics, and flexible therapeutic strategies are always kept pace with patient’s individual characteristics. Further studies including large samples focusing on cuspid morphology, periodontal condition, and different skeletal patterns are needed to explore the relationship between cuspids and alveolar bone.

## Conclusions

Our study demonstrated the characteristics of cuspid roots cortical anchorage and the factors influence the relationship between cuspid root and alveolar bone cortex in different sagittal skeletal patterns using 3D images, which provides a reference for clinicians to better implement orthodontic treatment and reduce complications. Importantly, 6.03 ± 4.41° (male) and 6.08 ± 4.45° (female) may be the appropriate root control angles to keep the roots of maxillary cuspids detached from the labial bone cortex. However, we only focused on normodivergent patients. Future studies on cuspids including large samples with different skeletal patterns are needed to enhance clinical treatment.

## Data Availability

The datasets generated and analyzed during the current study are not publicly available due to individual privacy but are available from the corresponding author on reasonable request.

## List of Abbreviations

CBCT: cone-beam computed tomography
CEJ: cemento-enamel junction
FMA: Frankfort mandibular plane angle
